# Comparative Safety and Efficacy of Left Atrial Appendage Occlusion with the Watchman Device and Amplatzer Cardiac Plug: Results of the Russian National Registry

**DOI:** 10.1155/2020/2352648

**Published:** 2020-11-07

**Authors:** Karapet Davtyan, Georgiy Simonyan, Arpi Topchyan, Andrey Kalemberg, Alexander Romanov, Vitaliy Shabanov, Dmitriy Lebedev, Sergey Gureev, Yulia Miller, Evgeniy Merkulov, Dmitry Pevzner, Pavel Mozgovoy, Vladimir Ufimtsev, Sergey Boytsov, Oksana Drapkina

**Affiliations:** ^1^National Medical Research Center for Therapy and Preventive Medicine, 101990 Moscow, Russia; ^2^E. Meshalkin National Medical Research Center, 630055 Novosibirsk, Russia; ^3^Almazov National Medical Research Centre, 197341 Saint Petersburg, Russia; ^4^National Medical Research Center of Cardiology, 121552 Moscow, Russia; ^5^Clinic №1 at Volgograd State Medical University, 400079 Volgograd, Russia

## Abstract

**Purpose:**

This multicenter, prospective registry evaluated the comparative safety and efficacy of left atrial appendage occlusion (LAAO) using the Watchman device (WD) and the Amplatzer Cardiac Plug (ACP) in patients with nonvalvular atrial fibrillation (NVAF) in real-world clinical practice in Russia.

**Methods:**

The study included data from 200 consecutive NVAF patients (66.8 ± 7.8 years, 44.5% female, median CHA2DS2VASc 4, median HAS-BLED 3) who had undergone LAAO implantation using WD (*n* = 108) or ACP (*n* = 92) from September 2015 to December 2017 in 5 medical centers in Russia. The primary safety endpoint was the procedure-related major adverse events, and the primary efficacy endpoint was the composite of thromboembolic events, device thrombosis, hemorrhagic events, and unexplained death during the 12-month follow-up.

**Results:**

Successful LAAO was performed in all 92 (100%) patients with ACP and 105 (97.2%) with WD (*p* = 0.053). At 12 months, primary safety endpoint occurred in 6.5% of patients in the ACP group with no events in the WD group (6.5% vs. 0%, *p* = 0.008). During the 12-month follow-up, the primary efficacy endpoint has occurred in 8.3% of patients in the WD group (*n* = 9) and 1.1% of patients in the ACP group (*n* = 1) (*p* = 0.016).

**Conclusions:**

In this multicenter prospective registry, LAA closure with the WD was associated with significantly higher thromboembolic events rate in NVAF patients. Patients, receiving the ACP, had more procedure-related major adverse events. However, further multicenter studies are necessary to evaluate these findings.

## 1. Introduction

It is well known that ischemic stroke is the leading cause of mortality and disability in patients with atrial fibrillation (AF) [1]. Left atrial appendage (LAA) is the source of thrombus formation in more than 90% of patients with nonvalvular AF (NVAF) [2], and exclusion of LAA from systemic circulation may impede thrombus formation [3]. Several studies demonstrated that transcatheter LAA occlusion (LAAO) could be an alternative to oral anticoagulant therapy (OAC) in NVAF patients at high risk of thromboembolic (TEO) and hemorrhagic events [4].

Present devices for endovascular LAAO are divided into three categories according to the LAA closure principle: “plug,” “pacifier,” and “ligation” [4]. The plug principle is to obstruct the neck of LAA by the LAAO device lobe or umbrella, e.g., the Watchman device (WD) [5]. The pacifier devices have an additional disk to close LAA ostium from the left atrial site in addition to the device lobe/umbrella (Amplatzer Cardiac Plug (ACP), Amplatzer Amulet [6]). Complete ligation of the neck of the LAA is the mechanism of closure with ligation devices (LARIAT) [7].

However, the data on comparative safety and efficacy of LAA closure using WD and ACP are limited to a few single-center studies [8, 9].

This registry aimed at assessing the safety and efficacy of the WD compared with ACP for thromboembolic events prevention and adverse events in patients with NVAF in a multicenter study.

## 2. Methods

This multicenter, non-randomized prospective registry included data from 200 consecutive NVAF patients, who undergone LAAO implantation using WD (*n* = 108, WD group) or ACP (*n* = 92, ACP group) from September 2015 to December 2017 in 5 medical centers in Russia within the framework of the state catheter-based LAAO program. The WD (Boston Scientific, Natick, Massachusetts) and ACP (St. Jude Medical, St. Paul, MN, USA) were the only LAAO devices approved by the Federal Service on Healthcare Surveillance in Russian Federation. The registry protocol was initially approved by the National Ethics Committee and after that by each center's Local Ethics Committee. Written informed consent was obtained from all patients before enrollment.

The inclusion criteria were age ≥ 18 years, NVAF, CHA_2_DS_2_VASc ≥ 2, high risk of hemorrhagic events, and non-compliance with pharmacological OAC therapy. The exclusion criteria were significant mitral valve disease, left ventricular ejection fraction (LVEF) < 35%, the tendency to systemic thrombosis, and severe comorbidities.

Preprocedural transesophageal echocardiography (TEE) was performed to exclude LAA thrombosis and evaluate LAA dimensions. LAAO procedures were carried out under continuous intravenous sedation with propofol or general anaesthesia with endotracheal intubation. Transseptal puncture was performed under the TEE guidance using an 8 Fr. transseptal sheath (usually SL0, St. Jude Medical) with the Brockenbrough needle. Intravenous heparin was administered at the time of transseptal puncture (target activated clotting time ≥ 250 s). A pigtail catheter was advanced over the 0.035-inch J-tipped guidewire into the LAA, and LAA angiography was performed in several views for better evaluation of LAA anatomy. The device size was selected to be 10-20% larger than the diameter of the landing zone based on TEE and angiographic measurement of LAA [4]. After that, a stiff J-tipped guidewire was inserted either into the distal LAA or left superior pulmonary vein depending on the operator. The transseptal sheath was exchanged to the appropriate delivery system, and the occluder was advanced via the delivery system to the LAA. After deployment, the device position, compression grade, and the completeness of LAA closure were assessed by both TEE (including colour-flow Doppler) and angiography. Tug test under fluoroscopic and TEE guidance was performed to confirm device stability [4, 6].

In two clinics, either WD or ACP was available for LAAO implantation. In the other three clinics, both WD and ACP were used, and device selection depended on operator preferences.

Postprocedural antithrombotic therapy strategy was left to the discretion of doctors.

Patients' baseline characteristics, procedure, and follow-up data were collected according to established registry protocol. Patients were followed at 45 days and 3, 6, and 12 months after enrollment. Diagnostic TEE was performed at 45 days and six months to assess LAAO device positioning, smoothness of atrial surface, and presence/absence of residual leaks. At each follow-up visit, the data regarding clinical events and healthcare utilization was collected.

The primary safety endpoint included procedure-related major adverse events (pericardial effusion, cardiac tamponade, device embolization, and procedure/device-related death). The primary efficacy endpoint was the composite of TEO, device thrombosis, hemorrhagic events, and unexplained death during the 12-month follow-up. The secondary endpoints were successful LAAO implantation rate and incidence rates of significant (≥5 mm) leakage on 6-month TEE.

### 2.1. Statistical Analysis

The number of study participants (*n* = 200) was predefined by the government-funded character of the trial. Continuous variables were presented as mean ± standard deviation (SD), median (Me), and interquartile range (IQR), categorical variables—as frequencies. Comparisons between two groups were performed with the Mann–Whitney *U* test, chi-square test, and 2-sided Fisher's exact test, as appropriate. A Spearman's correlation test was performed to evaluate the relationship between two methods for measuring LAA size. The Kaplan-Meier analysis with the log-rank test was run to estimate primary endpoints. The Cox proportional hazard model was used to evaluate the effect of prior LAAO procedure experience on the primary endpoints when possible: the hazard ratio (HR) and 95% confidence intervals (CI) were calculated. A two-tailed *p* value ≤ 0.05 was regarded significant. Statistical analysis was performed using Stata v15.0 for Windows (StataCorp., USA).

## 3. Results

### 3.1. Patients' and Procedural Characteristics

Two hundred patients with NVAF from 5 medical centers, who underwent LAAO implantation using WD (*n* = 108) and ACP (*n* = 92), were consecutively recruited in the study between September 2015 and December 2017. The mean age of the total study participants was 66.8 ± 7.8 years; there were slightly fewer females (44.5%) than males. No significant differences were observed in age (66.9 ± 7.9 vs. 66.7 ± 7.6, *p* = 0.795) and gender (female 45.4% vs. 43.5%, *p* = 0.795) between the groups. The percentage of patients with concomitant arterial hypertension (AH) was significantly higher in WD group (90.7% vs. 76.4%, *p* = 0.006), the frequency of congestive heart failure (CHF) (59.3 vs. 80.4, *p* = 0.001), and prior ischemic stroke (19.4 vs. 32.6, *p* = 0.033)—in ACP group. The CHA_2_DS_2_VASc score ranged from 2 to 8 (median score 4). The median of HAS-BLED score was 3. More than half of the patients were taking Warfarin, but only the third of them achieved the target INR (32.3%).

Baseline clinical and demographic characteristics of patients are presented in Table 1.

The same operators carried out the LAAO procedures in each clinic. Successful LAA occluder implantation was performed in all 92 (100%) patients with ACP and 105 (97.2%) with WD (*p* = 0.053). In these 3 cases, there was a displacement of the WD during tug test despite several attempts of reposition. The mean size of WD was 26.3 ± 3.8 mm, and the mean ACP size was 24.6 ± 3.7 (*p* = 0.002). There was no significant difference in the mean contrast media volume used (155 vs. 150 ml, *p* = 0.776).

### 3.2. Primary Endpoints

188 from 200 patients (94%) completed a 12-month clinical follow-up. One procedure-related death occurred the next day after the procedure. Two patients died during the follow-up period. One of them in the ACP group died in 6 weeks after LAAO implantation. No autopsy was performed; therefore, the exact cause of death was not determined. The other patient in the WD group died in 8 months after the procedure from stomach cancer.

**Figure 1:**
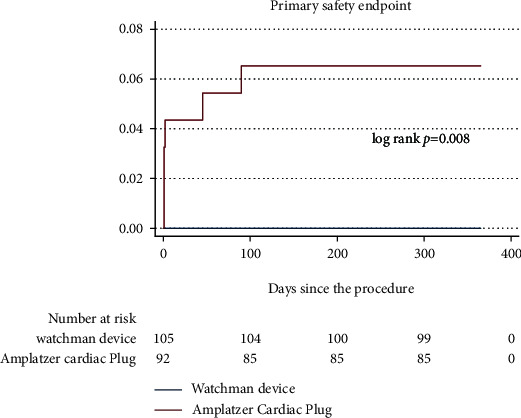
Major LAAO-related complications rate using the Watchman device and Amplatzer Cardiac Plug.

At 12 months, primary safety endpoint occurred in 6.5% of patients in the ACP group with no events in the WD group (6.5% vs. 0%, *p* = 0.008) (Figure 1).

In the ACP group, one procedure-related death occurred the next day due to pulmonary trunk perforation with fatal cardiac tamponade, and one patient developed haemotamponade requiring pericardiocentesis. In one case, device migration occurred to the left ventricle the next day, and subsequent endovascular removal of the occluder device was performed. In two patients, device embolization to abdominal aorta was identified on 45-day TEE. In one case, LAAO device was successfully removed via the right femoral artery by the percutaneous procedure. The other patient underwent open aortic surgery with aorto-left femoral bypass. One patient in the ACP group developed intraprocedural right femoral artery trauma by LAAO device sheath requiring urgent surgical intervention (Table 2).

Vascular access site minor complications (hematoma and bleeding), managed conservatively, occurred in one patient from each group. The procedure-related total complication rate was significantly higher in the ACP group compared with the WD group (9.8% vs. 0.9%, *p* = 0.006).

**Figure 2:**
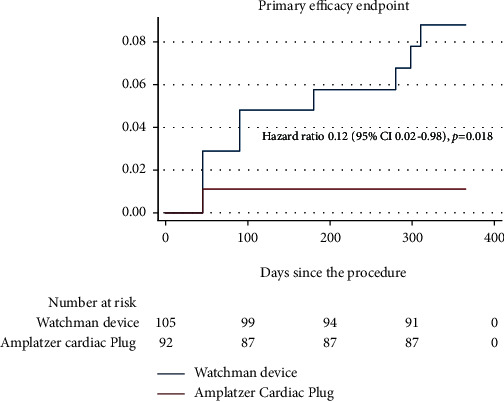
Primary efficacy endpoint of LAAO using Watchman device and Amplatzer Cardiac Plug.

At 12 months, the primary efficacy endpoint occurred in 8.3% of patients in the WD group (*n* = 9) and 1.1% of patients in the ACP group (*n* = 1) (*p* = 0.018) (Figure 2).

On 45-day TEE, silent thrombus was observed on the WD in three patients, who were treated with dual antiplatelet therapy (DAPT). In all cases, thrombus resolved on Enoxaparin sodium therapy (within 21 days) without any sequelae. TIA/stroke occurred in 5 patients (6.2%) in the WD group with no such events in the ACP group. Two patients presenting with stroke were taking DAPT, and the other two were being treated with NOAC and the combination of NOAC and Aspirin, respectively. One patient did not get any antithrombotic medications. Also, one patient in the WD group developed nasal bleeding requiring hospitalization.

### 3.3. Secondary Endpoints

There was no significant difference in LAAO device implantation success rate (97.2% vs. 100%, *p* = 0.053) and significant peridevice leakage (≥5 mm) incidence rate (3.0% vs. 0%, *p* = 0.059) between the WD group and ACP group. In two patients with leak, silent thrombus formation on the atrial side of WD was observed on 45-day TEE. Both thrombus cases were successfully managed with OAC.

### 3.4. LAAO Procedure Experience-Based Efficacy and Safety

The complications rate between centers ranged from 0% to 10.3% depending on the operators' experience. Only three operators had prior experience of catheter-based LAAO procedure with the implantation volume no less than 50, which was associated with a significantly lower complications rate (0.8% vs. 6.2%, *p* = 0.04). Five in six cases of major procedure-related adverse events occurred in two clinics without prior LAAO experience. Moreover, only ACP was available in one of these two clinics without prior LAAO experience. The frequency of the WD implantation in experienced centers was significantly higher (74.1% vs. 23.5%, *p* < 0.0001). However, there was no significant difference in the primary efficacy endpoint depending on center LAAO procedural volume (HR 1.27 (95% CI 0.24-6.6, *p* = 0.78)).

### 3.5. Postprocedural Antithrombotic Therapy Strategy

**Figure 3:**
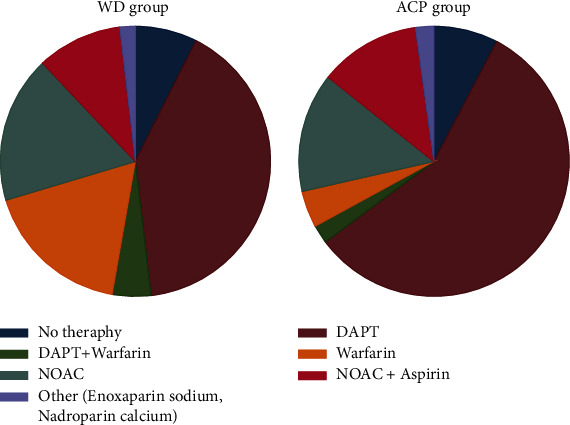
Antithrombotic therapy strategy after LAAO procedure. DAPT, dual antiplatelet therapy; NOAC, non-Vitamin K antagonist oral anticoagulants.

Oral anticoagulation therapy strategy was left to the physicians' discretion. Antithrombotic therapy did not significantly differ from that in the ACP group (*p* = 0.054) (Figure 3). At 12-month follow-up, the rate of compliance with antithrombotic therapy in the WD group was comparable to that in the ACP group (92.5 vs. 92.1%, *p* = 0.934). Approximately half of the patients were on DAPT, and third of the patients on anticoagulant therapy (Warfarin or NOAC). 14.6% of patients were taking a combination of oral anticoagulants with antiplatelets, and 2.0% of patients were taking injective anticoagulants (Enoxaparin sodium, Nadroparin calcium).

## 4. Discussion

We evaluated the safety and efficacy of the two most frequently used LAAO devices in Russia (the WD and ACP) in real-world clinical practice. The major findings of this study were that ACP implantation was associated with greater major complications' rate and the use of WD—with higher device thrombus formation/TIA/stroke rate.

The observed major adverse event rate in the ACP group (6.5%) was higher than that reported in the large multicenter trial with the Amplatzer Cardiac Plug (*n* = 1047) by Tzikas et al. (4.97%) [10]. Such high rate is most likely related to the concept of the learning curve [11, 12]. This government-funded catheter-based LAAO program in Russia started in September 2015, and a certain lack of experience existed at that time; two of five clinics had no previous experience of LAAO procedures. Moreover, the frequency of the WD implantation in experienced centers was significantly higher. In the study, conducted by Cruz-Gonzalez et al. [11] to assess the significance of the learning curve in LAAO, the complication rate decreases from 9% to 0% with operator experience. Moreover, the authors noticed that initial experience of LAAO using ACP also translated into better safety and efficacy outcomes for the WD [11]. The absence of additional disk in WD perhaps simplified the implantation procedure using the WD and therefore the complication rate [12]. In our study, no major adverse events occurred in the WD group, which correlated well with data from the EWOLUTION registry [13]. The analysis of two European real-world registry data, performed by Kleinecke et al. [14] reported a comparable periprocedural major complications rate for the WD and Amplatzer group devices (4.1% vs. 6.0%, *p* = 0.32). A recent meta-analysis of six studies also confirmed the comparable safety outcomes for the WD and Amplatzer groups [15].

Accurate LAAO device size selection is crucial to prevent significant peridevice leakage, which seems to be more common for the WD. In our study on 6-month TEE, major residual leakage was observed in 3 patients. Two of them developed devices' thrombi, fortunately, resolved without any clinical sequelae.

Data regarding the clinical efficacy of LAAO procedure for preventing stroke/TIA correlated strongly with respective studies for the WD [16] and ACP [10]. Tzikas et al. [10] reported the rate of 3.5% of TIA/stroke/cardiovascular death for an average 13-month follow-up period. In our study, sudden death occurred in 1 patient (1.1%). Although the number of patients with prior stroke history was significantly higher in the ACP group, there were no clinically significant TIA/stroke episodes in this group. The possible explanation of such findings is the results of a study, conducted by Litwinowicz et al. [17]. The authors evaluated the long-term efficacy of LAAO procedure in primary and secondary stroke prevention and reported comparable efficacy outcome during a mean follow-up of 50 months. The 6.2% rate of TIA/stroke in the WD group, observed in our study, correlated well with data from the PREVAIL trial (6.4%) [16] and the aforementioned European registries (6.4%) [14].

There was no significant difference in comparative efficacy and safety outcomes of LAA closure with WD versus ACP presented by Figini et al. [9]. Peridevice leak incidence was the only significant difference between the groups (WD 18% vs. ACP 6.3%, *p* = 0.037). Such a high incidence of the leak in the WD group was due to cutoff value > 3 mm for the severe residual leak. These data correlated well with the results of the aforementioned meta-analysis [15]. The rate of total peridevice leakage was significantly higher in the WD group (log OR = 1.32; 95%CI = 0.76 to 1.87; *p* < 0.01). On the other hand, the rate of significant peridevice leakage did not differ significantly between groups. All-cause mortality, cardiovascular mortality, and TIA/stroke rates were also comparable between the WD and Amplatzer device groups [15]. Direct comparison of three devices, the WD, ACP/Amulet, and LAmbre, performed in China, also did not reveal any statistically significant differences in procedure-related events and long-term follow-up outcomes [8]. However, the Amplatzer group included ACP and Amulet devices, which may lead to certain misinterpretation. LAAO procedure performed in a single center by more experienced operators might be the possible causes of such discrepancies between our results and reported data.

## 5. Limitations

This study is a retrospective analysis of prospectively collected registry data from a limited number of study participants. Also, the interpretation of the data was limited due to the small number of events. The device selection depended on operators' discretion and device availability: in two clinics, either the WD or ACP was available. Postprocedural antithrombotic therapy strategy was left to the discretion of treating physicians, which impedes the analysis of postprocedural antithrombotic therapy. However, this is the first study based on the multicenter prospective registry providing real-world experience with LAAO device.

## 6. Conclusion

In this multicenter prospective registry, LAA closure with the WD was associated with significantly higher thromboembolic events rate in NVAF patients. Patients, receiving the ACP, had more procedure-related major adverse events. However, further randomized multicenter studies are necessary to evaluate these findings.

## Figures and Tables

**Table 1:** Patients characteristics.

	Total	WD	ACP	*p* value
Age (years)	66.8 ± 7.8	66.9 ± 7.9	66.7 ± 7.6	0.795
Female gender % (*n*)	44.5 (89)	45.4 (49)	43.5 (40)	0.788
Permanent AF % (*n*)	46.5 (93)	50 (54)	42.4 (39)	0.539
AH % (*n*)	84.3 (166)	90.7 (98)	76.4 (68)	0.006
DM % (*n*)	21(48)	25.9(28)	21.7 (20)	0.490
Prior MI % (*n*)	18.1 (36)	19.6 (21)	16.3 (15)	0.544
CHF 2-3 NYHA % (*n*)	69.0 (138)	59.3 (64)	80.4 (74)	0.001
Prior ischemic stroke % (*n*)	25.5 (51)	19.4 (21)	32.6 (30)	0.033
Prior hemorrhagic stroke % (*n*)	4 (8)	4.6 (5)	3.3 (3)	0.622
TIA % (*n*)	6 (12)	6.5 (7)	5.4 (5)	0.756
Neurologic deficiency % (*n*)	17.1 (34)	13.1 (14)	21.7 (20)	0.106
Major bleeding % (*n*)	39.4 (78)	33.3 (36)	46.7 (42)	0.056
Carotid artery stenosis % (*n*)	6 (12)	3.7 (4)	8.7 (8)	0.138
Hepatic dysfunction % (*n*)	2.0 (4)	0.9 (1)	3.3 (3)	0.337
Interatrial shunting % (*n*)	9.7 (19)	12.0 (13)	6.8 (6)	0.219
Alcohol abuse % (*n*)	1.0 (2)	0.9 (1)	1.1 (1)	0.909
OAC % (*n*)				0.198

Warfarin	62.6 (124)	66.7 (72)	57.8 (52)	

NOAC	37.4 (74)	33.3 (36)	42.2 (38)	
Labile INR % (*n*)	67.7 (84)	65.3 (47)	71.1 (37)	0.490
CHA_2_DS_2_VASc score	4 (3-5)	3.5 (3-5)	4 (3-5)	0.193
HAS-BLED score	3 (3-3)	3 (2-3)	3 (3-3)	0.348
LA size (mm)	47 (42-52)	47 (42-51.5)	47 (41-52)	0.735
LVEF (%)	57 (51-61)	58 (52-60)	55 (50-63,5)	0.325

Values are presented as mean ± standard deviation, % (*n*), or median (interquartile range). AF, atrial fibrillation; AH, arterial hypertension; DM, diabetes mellitus; MI, myocardial infarction; CHF, congestive heart failure; TIA, transient ischemic attack; OAC, oral anticoagulant therapy; NOAC, non-Vitamin K antagonist oral anticoagulants; INR, international normalized ratio; LA, left atrium; LVEF, left ventricular ejection fraction.

**Table 2:** Major complications of LAAO procedure.

Complication type	Number	Incident/detection time
Fatal pulmonary trunk perforation	1	1 day
Haemotamponade	1	0 day
Device migration to the left ventricle	1	1 day
Device migration to the aorta	2	45 day
Right femoral artery trauma	1	0 day

## Data Availability

The data that support the findings of this study are available from the corresponding author, upon reasonable request.
